# Assessment of COVID-19 vaccination among healthcare workers in Iraq; adverse effects and hesitancy

**DOI:** 10.1371/journal.pone.0274526

**Published:** 2022-11-18

**Authors:** Omeed Darweesh, Nasir Khatab, Ramiar Kheder, Thulfiqar Mohammed, Tola Faraj, Sabah Ali, Muath Ameen, Azad Kamal-Aldin, Mohammed Alswes, Naif Al-Jomah

**Affiliations:** 1 College of Pharmacy, Al-Kitab University, Kirkuk, Iraq; 2 Department of Molecular and Cell Biology, University of Leicester, Leicester, United Kingdom; 3 Department of Public Health, Kirkuk Health Directorate, Ministry of Health, Kirkuk, Iraq; 4 Medical Laboratory Science Department, College of Science, University of Raparin, Sulaymaniyah, Iraq; 5 Department of Medical Analysis, Faculty of Applied Science, Tishk International University, Erbil, Iraq; 6 National Institute of Technology, Sulaymaniyah, Iraq; 7 Department of Basic Sciences, College of Medicine, Hawler Medical University, Erbil, Iraq; 8 College of Pharmacy, Knowledge University, Erbil, Iraq; 9 Department of Family Medicine and Policlinics, Research Centre, King Faisal Specialist Hospital and Research Centre, Riyadh, Saudi Arabia; 10 Molecular Oncology Department, Research Centre, King Faisal Specialist Hospital and Research Centre, Riyadh, Saudi Arabia; University of South Australia, AUSTRALIA

## Abstract

Several messenger ribonucleic acid (mRNA) and inactivated COVID-19 vaccines are available to the global population as of 2022. The acceptance of the COVID-19 vaccine will play a key role in combating the worldwide pandemic. Public confidence in this vaccine is largely based on its safety and effectiveness. This study was designed to provide independent evidence of the adverse effects associated with COVID-19 vaccines among healthcare workers in Iraq and to identify the attitudes of healthcare workers who rejected the vaccination. We conducted a cross-sectional study to collect data on the adverse effects of the Pfizer, AstraZeneca, and Sinopharm vaccines. Data were collected between October 2021 and February 2022. A total of 2,202 participants were enrolled in the study: (89.97%) received injections of the COVID-19 vaccines and (10.03%) were hesitant to receive the vaccination. Participants received either the Pfizer vaccine (62.9%), AstraZeneca vaccine (23.5%) or Sinopharm vaccine (13.6%). Most adverse effects were significantly less prevalent in the second dose than in the first dose. Notably, the adverse effects associated with the Pfizer vaccine were significantly more prevalent in females than in males. Following the first dose, the participants experienced more adverse effects with the AstraZeneca vaccine. Following the second dose, more adverse effects were associated with the Pfizer vaccine. Interestingly, the prevalence of COVID-19 infection in participants who received two doses of the Pfizer vaccine was significantly reduced compared to those who received two doses of either the AstraZeneca or Sinopharm vaccines. According to vaccine-hesitated participants, insufficient knowledge (29.9%), expeditious development (27.6%) and lack of trust in the vaccines (27.1%) were the three major reasons for refusing the vaccines. The results of our study indicated that these adverse effects do not present a significant problem and should not prevent successful control of the COVID-19 pandemic.

## Introduction

As there is no approved antiviral treatment for COVID-19 infection, vaccination is the most effective intervention for combating the disease [1]. Vaccination prevents symptomatic COVID-19 infection and minimizes the risks of severe illness, by stimulating the immune system to produce antibodies [2–4]. Trials to develop vaccines were immediately initiated in the hope of controlling this pandemic. The first vaccines approved by global health authorities were the Pfizer-BioNTech mRNA vaccine (BNT162b2) and the Oxford-AstraZeneca vaccine (ChAdOx1 nCoV-19), followed by Sinopharm (BBIBP-CorV) [5]. BNT162b2 is a vaccine based on mRNA coding for SARS-CoV-2 spike protein that has demonstrated 95% efficacy against symptomatic COVID-19 infection [6]. AstraZeneca developed ChAdOx1 nCoV-19, a replication-deficient chimpanzee adenovirus particle expressing the full length of the spike protein. The AstraZeneca vaccine was authorized to be used in the age group of 18 years and older and showed 66% efficacy against COVID-19 infection [7]. In contrast, China developed the Sinopharm vaccine, which consists of an inactivated strain of SARS-CoV-2 HB02 with an efficacy of 79% [5].

There is no vaccine that is completely free of adverse effects. Vaccination confers immunity against COVID-19, regardless of whether adverse effects occur or not [8]. Potential postvaccine adverse effects are thought to be the primary reason for vaccine hesitancy. Improving vaccine acceptance requires increasing public awareness of vaccine efficacy and being honest about adverse effects [9, 10]. Vaccine adverse effects depend on the type of vaccine: for example, mRNA vaccines are associated with higher postvaccination adverse effects than other vaccines [11, 12]. A large-scale study indicates that pain at the injection site (58%) presented alongside fatigue (46%), headache (45%), fever (39%), joint pain (38%) dizziness (28%) and chills (28%) are the most common side effects after COVID-19 vaccines [12].

The COVID-19 vaccine is more likely to be given to adults. Moreover, healthcare workers are more cautious about getting vaccinated because of the nature of their work and their exposure to COVID-19 patients [7]. Based on the medical and scientific background of the study sample, a major strength of this study is that it was based on healthcare professionals who were expected to provide accurate and transparent information. Although the epidemiology and outcome of COVID-19 have been progressively studied [13–15], there is a lack of knowledge about COVID-19 vaccines efficacy and adverse effects in different countries [16]. The efficacy and adverse effects of mRNA and inactivated vaccines against COVID-19 continue to be studied, primarily in Europe and the United States [17–20]. A few studies have been conducted in Jordan, Saudi Arabia, the UAE, and Egypt [8, 21–24]. Therefore, in this study we aimed to compare the adverse effects of COVID-19 vaccines among healthcare workers in Iraq. Three vaccines are currently authorized for use in Iraq: BNT162b2 (Pfizer), ChAdOx1 (AstraZeneca), and BBIBP-CorV (Sinopharm). Furthermore, we studied healthcare workers’ attitudes toward vaccination, especially among those who hesitate to receive the vaccine at present.

## Methods

### The study design

This observational, cross-sectional, survey-based, multicentre study was carried out from October 2021 to February 2022. An online questionnaire was created using Google Forms tools hosted at Al-Kitab University/Ministry of Higher Education and Scientific Research. The study was reviewed and approved by the ethics committee of the Faculty of Pharmacy/Al-Kitab University/Ministry of Higher Education and Scientific Research and Kirkuk health Directorate/Ministry of Health (Study ID REF 395 in 05/09/2021).

The self-administered survey was divided into six mandatory sections. The first part collected demographic information about the participants, including the county they lived in, age, gender, and profession. In the second part, healthcare workers were surveyed to determine whether they had decided to take the vaccine or not. Parts 3 and 4 of the survey asked participants to select any symptoms they had after the first and second doses of the vaccine, respectively. The participants were also asked to report other unlisted adverse effects. In the fifth section, the participants were asked several questions, including the type of vaccine they had received, whether they were previously infected with COVID-19 and how many times, and their medical history comprising comorbidities and medications. Female participants were asked whether they were pregnant when vaccinated or if they had a positive test for COVID-19 while pregnant. In the last section of the survey, we assessed the perceptions of vaccine rejectors and sources of information about COVID-19 vaccines.

The inclusion criteria for this study were healthcare workers, 20 years of age and older, living in one of these four counties and working in healthcare institutions, regardless of their role in directly dealing with COVID-19 patients. The eligible participants included those who received one dose or two doses of either Pfizer, AstraZeneca or Sinopharm vaccines and those who rejected vaccination. We excluded incomplete responses.

### Sampling technique

According to the Ministry of Health (Iraq), approximately 100,000 people are employed in hospitals and other healthcare institutions at the study site. We calculated a minimum representative sample size of 660 according to the Raosoft online sample size calculator, with a 5% margin of error, 99% confidence interval, and 50% response distribution. However, a total of 2,202 participants were enrolled in this study, which represents more than three-fold of the required sample size. Our study sample was increased to strengthen the statistical analysis.

### Data collection

Data were collected anonymously from the participants, with no personal identification. In addition to being distributed via administrative leaders at main hospitals and dental clinics in four different counties, Kirkuk, Erbil, Mosul and Baghdad, the survey tool was distributed with links on social media platforms (Facebook, WhatsApp, and email) to healthcare worker groups. A consent form was obtained from each participant prior to recruitment.

### Data analysis

The data collected in Google Forms were exported into a Microsoft Excel file, which was imported directly into a GraphPad prism (version 9.0) for statistical analysis. Significant differences were determined using a Chi-squared test, as shown in the figure legends, and are indicated with one asterisk (*P < 0.05), double asterisks (**P < 0.01), three asterisks (***P < 0.001), or four asterisks (****P < 0.0001).

## Results

### Characteristics of the participants

**Fig 1:**
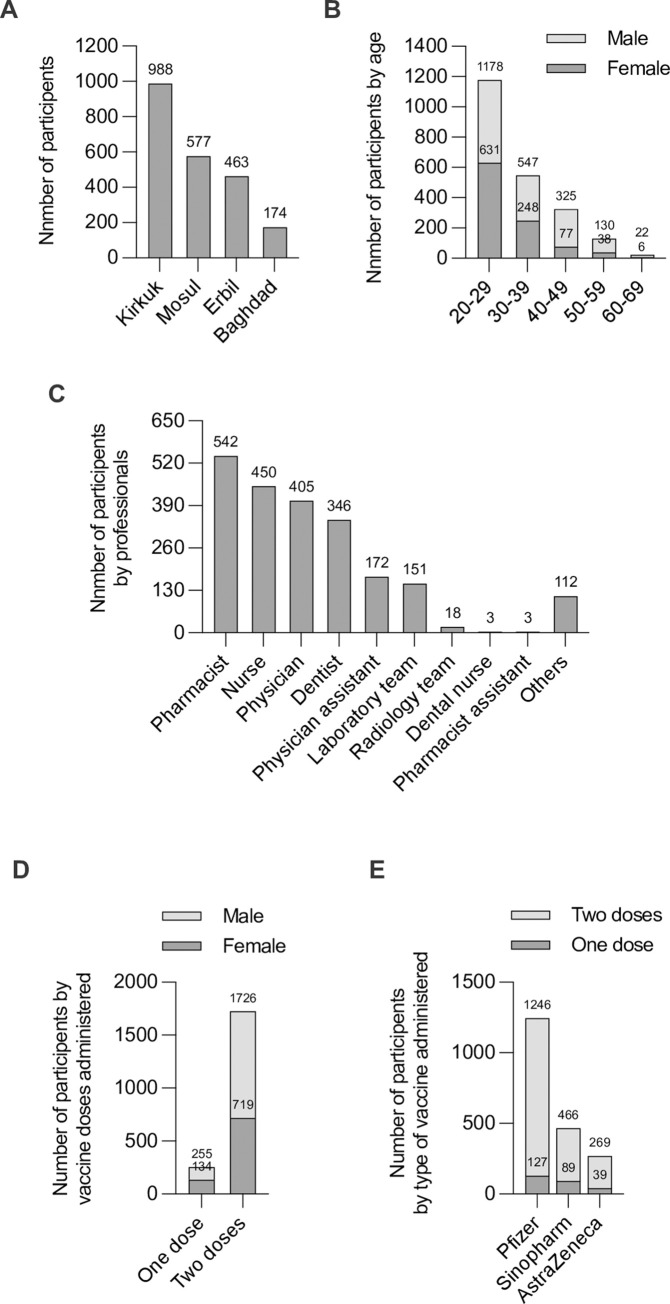
Demographic characteristics of the healthcare workers who participated in the study. (A) Number of participants by counties, (B) Number of participants by age groups, (C) Number of participants by professionals, (D) Number of participants by vaccine doses administered, (E) Number of participants by type of vaccine administered.

A total of 2,202 healthcare workers were included in the study: 1,204 (54.7%) were males and 998 (45.3%) were females. Of them, 1,981 (89.97%) had received injections of the COVID-19 vaccines, which represents the acceptance rate among healthcare workers at the study site, and 221 (10.03%) had been hesitant about the vaccination. Participants were from four counties: Kirkuk 988 (44.9%), Mosul 577 (26.2%), Erbil 463 (21%) and Baghdad 174 (7.9%) (Fig 1A). Based on age, the participants were divided into five main groups. The results showed that most participants were in the age range of 20–29 years old (53.5%), 30–39 years (24.8%), 40–49 years (14.8%), 50–59 years (5.9%), and 60–69 years (1%); the mean age was 32.44 ± 9.34 years, and ranged from 20 to 69 years (Fig 1B). The study sample consisted of a variety of healthcare professionals; 542 (24.6%) pharmacists, 450 (20.5%) nurses, 405 (18.4%) physicians, 346 (15.7%) dentists, 172 (7.8%) physician’s assistant, 151 (6.9%) laboratory team members, 18 (0.8%) radiology team members, three dental nurses and pharmacist assistants each (0.1%), and 112 (5.1%) administrative staff members at healthcare facilities without direct patient care (Fig 1C). At the time of completing the questionnaire, the majority of the participants 1,726 (87.1%) had received two doses of COVID-19 vaccines, while 255 (12.9%) had received the first dose only (Fig 1D). With regard to the type of COVID-19 vaccine, the Pfizer vaccine was administered to a large proportion of the study population (1,246) (62.9%), while 466 (23.5%) received the AstraZeneca vaccine, and 269 (13.6%) received the Sinopharm vaccine (Fig 1E). Most of the participants (94.3%) reported having no chronic medical conditions, and only 5.7% had secondary diseases. The chronic medical conditions of the 1,981 vaccinated participants are presented in Table 1.

**Table 1:** Chronic medical conditions of vaccinated participants.

Chronic medical conditions	Pfizer n1 = 1247	AstraZeneca n2 = 466	Sinopharm n3 = 269	Study sample n = 1981
	(%)	(%)	(%)	(%)
**Allergic Bronchitis**	0.32	0	1.11	0.35
**Asthma**	0.96	0.42	3.71	1.21
**Hypertension**	0.56	0.42	2.23	0.75
**Diabetes Mellitus**	0.8	0.21	1.11	0.7
**Heart Disease**	0.4	0.21	1.85	0.55
**Breast Cancer**	0.08	0	0.37	0.1
**Urticaria**	0.24	0	0	0.15
**Arthritis**	0.08	0	0	0.05
**Inflammatory Bowel Disease**	0.08	0	1.48	0.25
**Systemic Lupus Erythematosus**	0.08	0.21	0	0.1
**Bipolar Disorder**	0	0.21	0	0.05
**Anxiety**	0.16	0	0	0.1
**Epilepsy**	0.24	0.21	0	0.2
**Hyperthyroidism**	0.24	0.21	0.37	0.25
**Hypothyroidism**	0.4	0.64	1.85	0.65
**Iron Deficiency Anaemia**	0.24	0	0	0.15
**Thalassemia**	0.08	0	0	0.05

### COVID-19 vaccine reported adverse effects

#### First and second dose adverse effects

To assess the adverse effects of the Pfizer, AstraZeneca and Sinopharm vaccines, data were collected from vaccinated healthcare workers. In the population analyzed, no serious adverse effects were recorded. None of the participants reported that they required hospitalization after the first or second dose of any of the COVID-19 vaccines. All reported adverse effects were non-serious, such as pain at the site of injection, muscle pain, headache, high temperature, joint pain, fatigue, tenderness, feeling sick, lethargy, redness at the site of injection, feeling achy, cough, nausea, pruritus at the site of injection, loss of taste or smell, and diarrhea.

**Fig 2:**
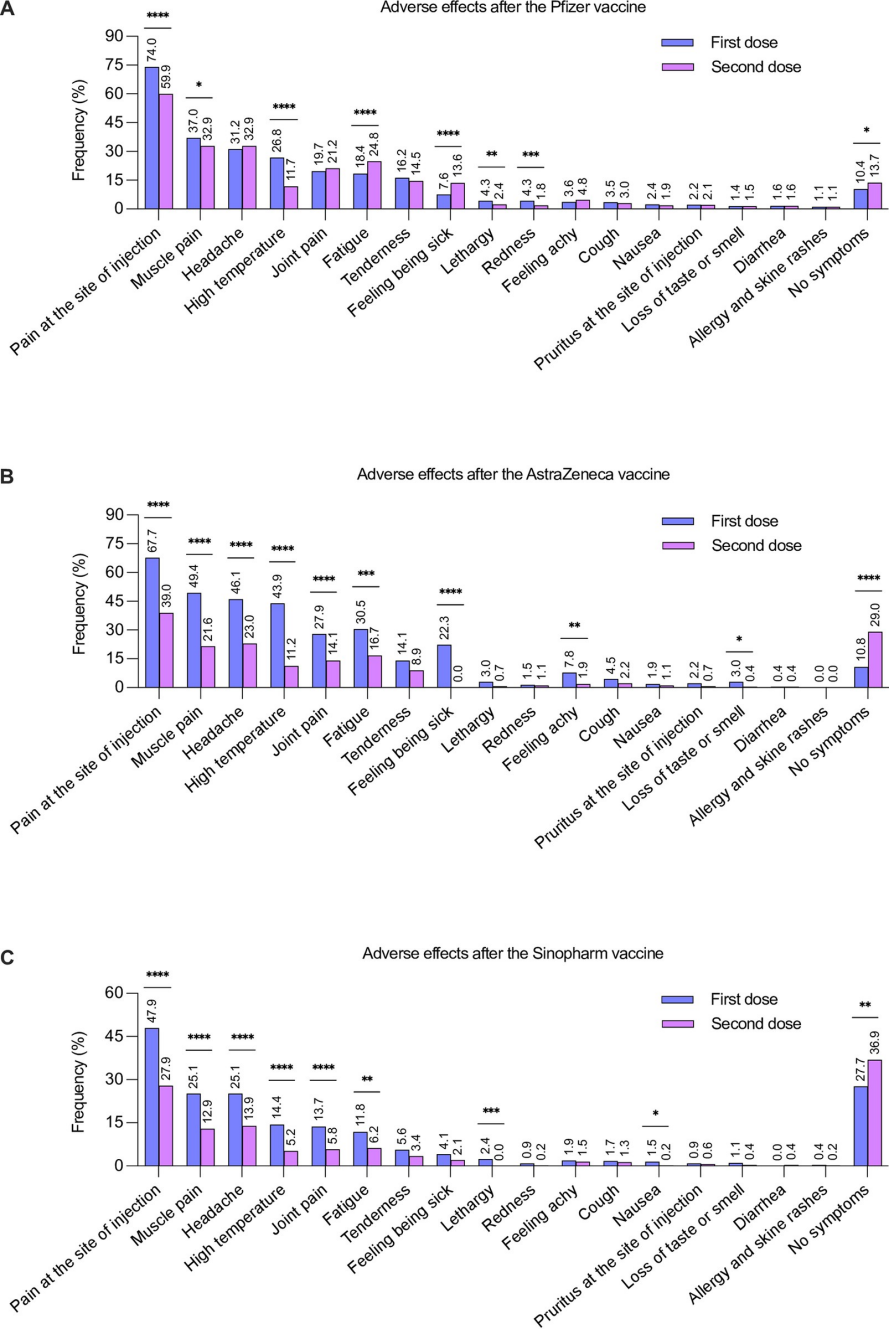
Prevalence of the adverse effects (percentage) associated with various vaccine types after the first dose and second dose. (A) Pfizer after the first and second dose, (B) AstraZeneca after the first and second dose, (C) Sinopharm after the first and second dose. Statistical analysis of the adverse effects associated with various vaccine types (first dose versus second dose) was performed using the chi-squared test. *p<0.05, **p<0.01, ***p<0.001 and ****p<0.0001.

The most common local adverse effect associated with the Pfizer vaccine was pain at the site of injection after both the first and second doses. On comparing the adverse effects that resulted from the first and second doses of the Pfizer vaccine, pain and redness at the site of injection were significantly more prevalent in the first dose compared to the second dose (Fig 2A). Among the systemic reactions, muscle pain, high temperature, and lethargy were the most common adverse effects. Their frequency was significantly higher in the first dose than in the second dose (Fig 2A). In contrast, fatigue and feeling sick after the second dose were more prevalent than after the first dose. On the other hand, 13.7% of those who received the second dose and 10.4% of those who received the first dose did not experience any adverse effects (Fig 2A).

Regarding the AstraZeneca vaccine, the most common adverse effect was pain at the site of injection, followed by muscle pain, headache, high temperature, joint pain, fatigue, tenderness, and feeling sick. First-dose adverse effects were statistically more prevalent than second-dose adverse effects. However, 29% of the participants who had the second dose and 10.8% of those who had the first dose reported no adverse effects (Fig 2B).

All adverse effects reported with the first dose of the Sinopharm vaccine were more prevalent than those reported with the second dose. However, 36.9% of the participants who had the second dose and 27.7% of those who had the first dose did not report any adverse effects (Fig 2C). Tables 2 and 3 show the less frequent short-term adverse effects caused by the first and second doses of COVID-19 vaccines, respectively.

**Table 2:** Other unlisted adverse effects associated with the first dose of COVID-19 vaccines.

Adverse effects	Pfizer	AstraZeneca	Sinopharm	Study sample
	n1 = 1246	n2 = 466	n3 = 269	n = 1981
	(%)	(%)	(%)	(%)
**Back pain**	0.24	0	0	0.15
**Chest pain**	0.08	0	0.37	0.1
**Abdominal pain**	0.08	0	0	0.05
**Stomach pain**	0.16	0	0.37	0.15
**Leg pain**	0	0.21	0	0.05
**Irregular heartbeat**	0	0.21	0.37	0.15
**Shortness of breath**	0.16	0	0.74	0.2
**Tremor**	0.16	0	0	0.1
**Dizziness**	0.24	0	0.74	0.25
**Vertigo**	0.08	0	0	0.05
**Depression**	0.08	0	0.37	0.1
**Nightmares**	0.16	0.42	0	0.2
**Sweating**	0	0.42	0	0.1
**Numbness or tingling**	0.16	0	0	0.1
**Mouth ulcer**	0	0	0.74	0.1
**Flu-symptoms**	0.08	0	0.37	0.1

**Table 3:** Other unlisted adverse effects associated with the second dose of COVID-19 vaccines.

Adverse effects	Pfizer	AstraZeneca	Sinopharm	Study sample
	n1 = 1119	n2 = 377	n3 = 230	n = 1726
	(%)	(%)	(%)	(%)
**Back pain**	0.8	0	0	0.52
**Chest pain**	0.08	0	0.43	0.11
**Abdominal pain**	0.08	0	0	0.05
**Stomach pain**	0.08	0	0.86	0.17
**Irregular heartbeat**	0.08	0	0.43	0.11
**Shortness of breath**	0.26	0.26	0	0.23
**Tremor**	0.17	0	0	0.11
**Dizziness**	0.17	0	0	0.11
**Vertigo**	0.08	0	0	0.05
**Depression**	0.35	0	0	0.23
**Nightmares**	0.08	0	0	0.05
**Sweating**	0	0.26	0	0.05
**Numbness or tingling**	0.17	0	0	0.11
**Mouth ulcer**	0	0	0.43	0.05
**Flu-symptoms**	0.08	0.26	0.43	0.17
**Swelling under the armpit**	0	0.26	0	0.05

#### Adverse effects among males and females

**Fig 3:**
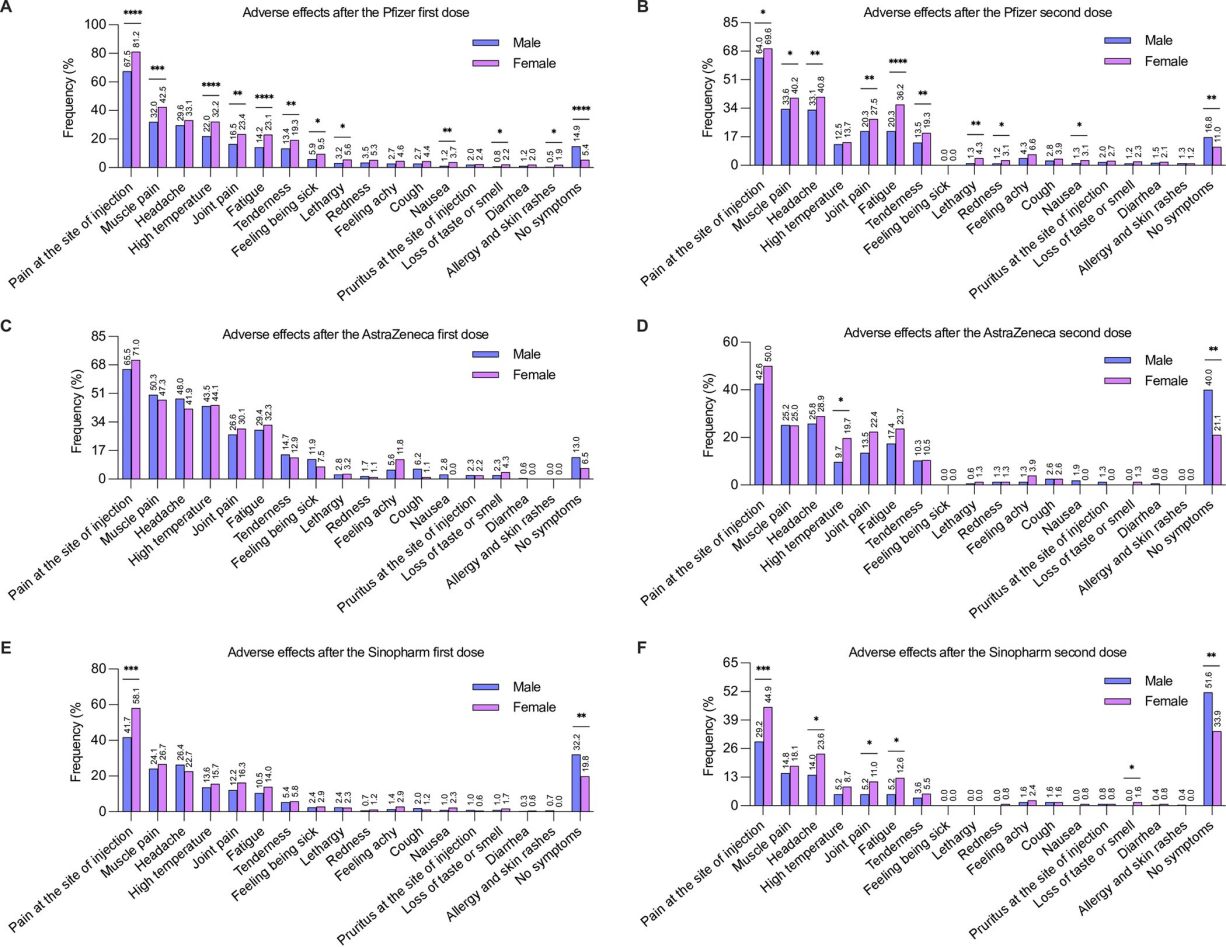
Prevalence of the adverse effects (percentage) associated with various vaccine types among vaccinated male and female participants. (A) Pfizer after the first dose, (B) Pfizer after the second dose, (C) AstraZeneca after the first dose, (D) AstraZeneca after the second dose, (E) Sinopharm after the first dose, (F) Sinopharm after the second dose. Statistical analysis of the adverse effects associated with various vaccine types (males versus females) was performed using the chi-squared test. *p<0.05, **p<0.01, ***p<0.001 and ****p<0.0001.

Next, we evaluated the adverse effects associated with COVID-19 vaccines among male and female participants. The common adverse effects associated with Pfizer’s first dose were significantly more prevalent in females than in males (Fig 3A). Additionally, the adverse effects that resulted from Pfizer’s second dose were significantly more prevalent among females than males (Fig 3B).

There was no significant difference in the adverse effects associated with AstraZeneca’s first dose between males and females. The most common symptoms reported by males and females were pain at the injection site (Fig 3C). Similar to the first dose, the second dose showed no significant differences, except for high temperature. In the chi-squared test, the prevalence of high temperature following the second dose was significantly higher in females than in males (Fig 3D).

Pain at the site of injection following Sinopharm’s first dose was significantly more prevalent in females than in males. However, the prevalence of other adverse effects associated with Sinopharm’s first dose was comparable, and no significant difference was detected between males and females (Fig 3E). In contrast, the prevalence of some adverse effects associated with Sinopharm’s second dose was significantly higher in females than in males (Fig 3F).

#### Comparing the adverse effects of Pfizer, AstraZeneca, and Sinopharm vaccines

**Fig 4:**
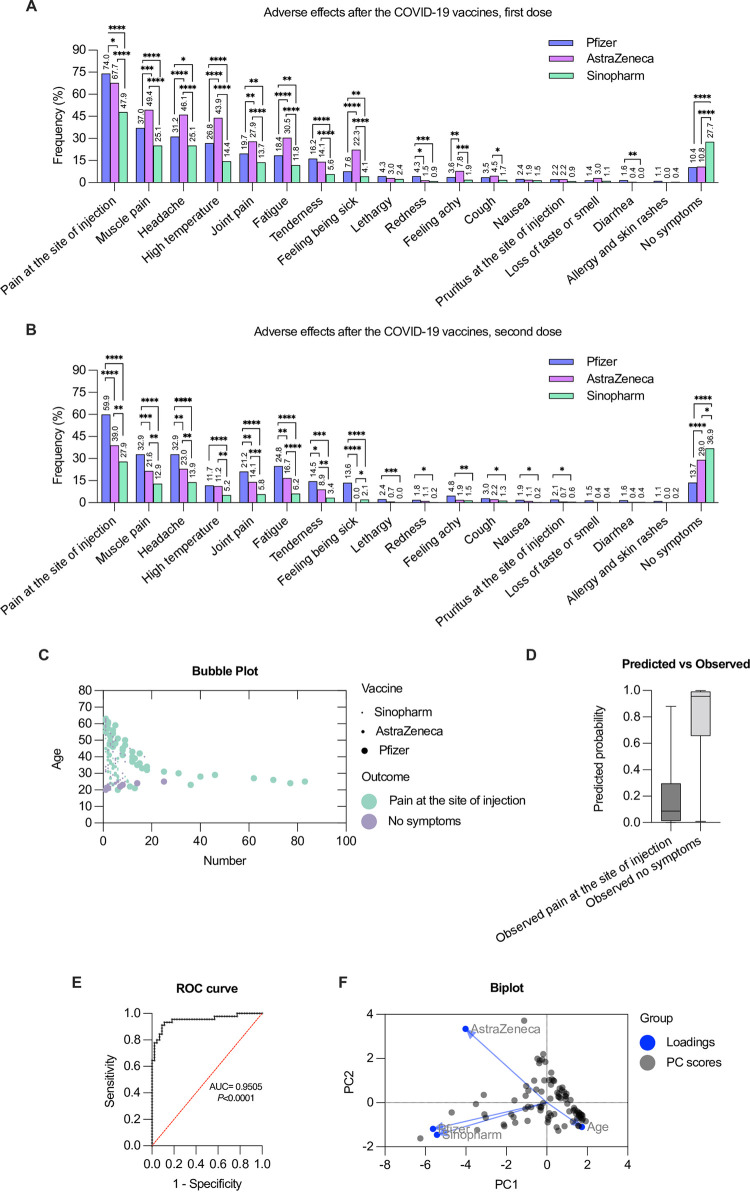
Prevalence of the COVID-19 vaccines adverse effects (percentage) associated with various vaccine types (Pfizer, AstraZeneca and Sinopharm) among vaccinated participants. (A) after the first dose, (B) after the second dose. A statistical analysis of the adverse effects associated with various COVID-19 vaccines (Pfizer versus AstraZeneca, Pfizer versus Sinopharm, AstraZeneca versus Sinopharm) was performed using the chi-squared test. *p<0.05, **p<0.01, ***p<0.001 and ****p<0.0001. (C) A bubble chart depicting the association between the significant outcomes of the vaccinated population, age and type of vaccine. (D) Predicted probability chart analysis using multiple logistic regression of results in (C). (E) ROC curve for the variables dependent outcomes using logistic regression indicating a better performance. Negative and positive predictive power were 90.91% and 91.11%, respectively for the cutoff point of 0.5. Area under the curve (AUC) and p value are shown. (F) Biplot showing the principal component scores and the loading vectors of the four characteristics. Plot shows the original variables as vectors (arrows).

Next, we compared the adverse effects associated with COVID-19 vaccines related to vaccine types following the first and second doses. Pain and redness at the injection site after the first dose of the Pfizer vaccine were significantly more prevalent than those of AstraZeneca and Sinopharm (Fig 4A). In contrast, most of the reported adverse effects associated with AstraZeneca’s first dose were significantly more prevalent than those associated with Pfizer or Sinopharm (Fig 4A). Interestingly, several adverse effects reported following the first dose of Sinopharm were significantly lower than those of either Pfizer or AstraZeneca (Fig 4A). Moreover, cough after the first dose was significantly more prevalent in AstraZeneca than in Sinopharm, and diarrhea was significantly more prevalent in Pfizer than in Sinopharm.

We examined the adverse effects caused by the different COVID-19 vaccines following the second dose. In comparison to AstraZeneca or Sinopharm, the Pfizer vaccines had a significantly higher rate of adverse effects (Fig 4B). Most of the adverse effects associated with AstraZeneca’s second dose were significantly more prevalent than those associated with Sinopharm (Fig 4B). However, feeling being sick was significantly higher in Sinopharm’s second dose than in AstraZeneca (Fig 4B).

The principal component analysis (PCA) was used to reduce the dimensionality of data sets. Since pain at the injection site is the most common adverse effect associated with COVID-19 vaccines, we generated a bubble chart to summarize the impact of vaccine type and age on this adverse effect (Fig 4C). This also indicates a high predicted probability of showing no symptoms (Fig 4D). A receiver operating characteristics (ROC) curve was generated to evaluate the performance of the classification. We found that the area under the curve (AUC) is 0.9505 with p<0.001, indicating the highest performance. (Fig 4E). In addition, Biplot analysis was used to show the scores and loadings in a single plot. The observation implies that there is a positive correlation between the Pfizer, Sinopharm, and AstraZeneca. While the age is in the opposite direction of the horizontal axis, meaning it has a negative loading value with respect to the other principal components (Fig 4F).

### Percentage of COVID-19 infection before and after vaccination

**Fig 5:**
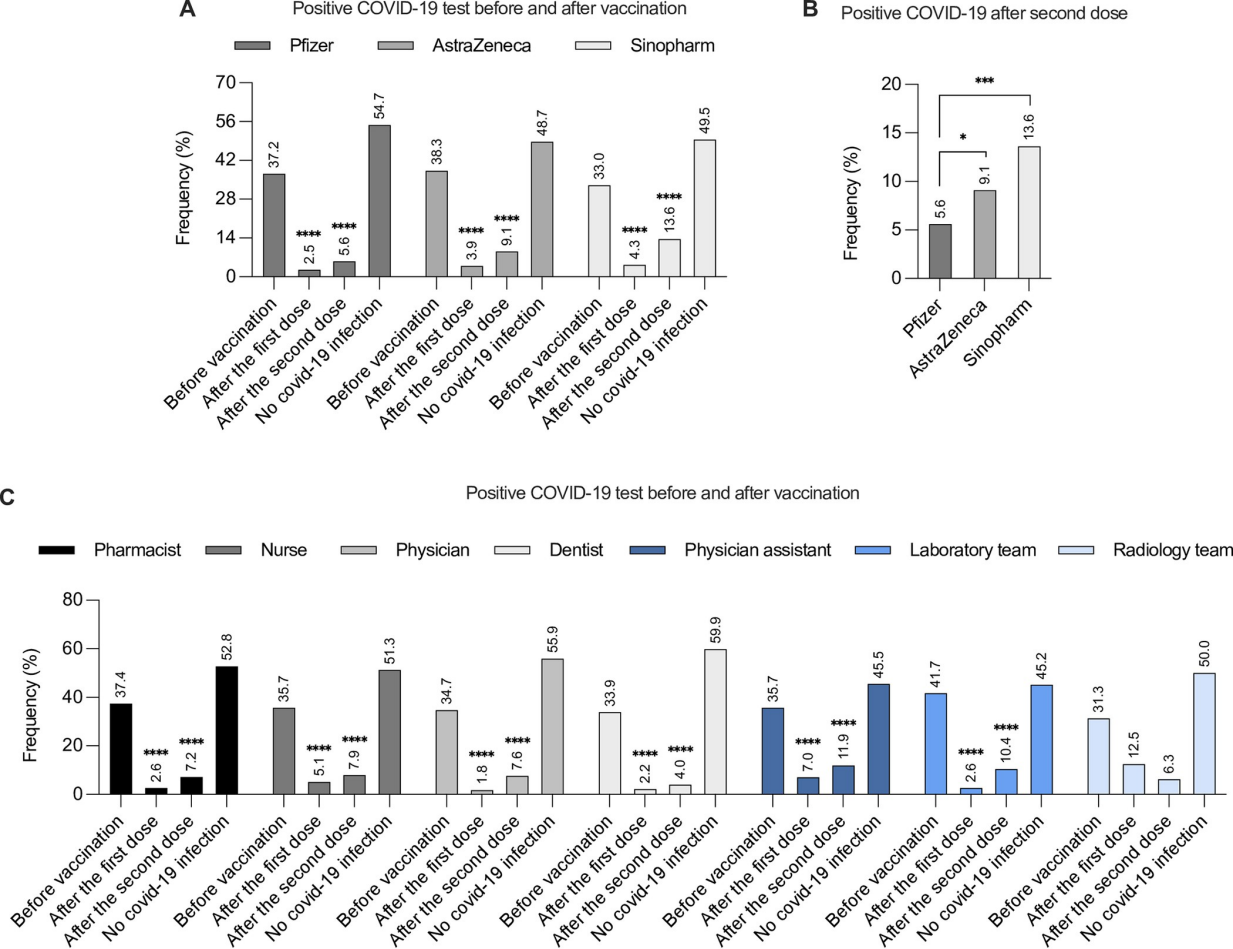
Prevalence of the COVID-19 infection (percentage) before and after vaccination. (A) prevalence of COVID-19 infection before, after one dose and after two doses of the Pfizer, AstraZeneca, and Sinopharm vaccines, (B) Prevalence of the COVID-19 infection after two doses of the Pfizer, AstraZeneca, and Sinopharm vaccines, (C) Prevalence of the COVID-19 infection among healthcare professionals before, after one dose and after two doses of the vaccines. A statistical analysis of the COVID-19 infection among vaccinated participants who received various vaccine types, (A) after the first dose and the second dose versus before vaccination, (B) after the second dose between various vaccine types or (C) among healthcare professionals, was performed using the chi-squared test. ****p<0.0001.

Our next step was to determine the percentage of COVID-19 infection before and after vaccination. A total of 1,246 participants received the Pfizer vaccine. Of them, 37.2% reported previous COVID-19 infection before receiving the vaccine, and 54.7% did not test positive for COVID-19. Both the first and second doses of the Pfizer vaccine resulted in a significant reduction in COVID-19 infection (2.5% and 5.6%, respectively) (Fig 5A). Regarding AstraZeneca, 466 subjects received the vaccine: 38.3% reported previous COVID-19 infection before being vaccinated and 48.7% were negative. The percentage of COVID-19 infections after the first and second doses of AstraZeneca vaccination was significantly reduced to 3.9% and 9.1%, respectively (Fig 5A). The Sinopharm vaccine was administered to 269 participants: 33% had COVID-19 infection before receiving the vaccine and 49.5% did not test positive. After receiving the first and second doses of Sinopharm, the proportion of COVID-19 infections was significantly reduced to 4.3% and 13.6%, respectively (Fig 5A). Interestingly, participants who received two doses of the Pfizer vaccine had a significantly lower COVID-19 infection rate compared to those who received two doses of the AstraZeneca or Sinopharm vaccines. (Fig 5B).

Regardless of their professions, healthcare workers had similar rates of COVID-19 infection before vaccination: pharmacists 37.4%, nurses 35.7%, physicians 34.7%, dentists 33.9%, physician assistants 35.7%, laboratory team members 41.7% and radiology team members 31.3% (Fig 5C). Nonetheless, vaccination resulted in a significant reduction in the proportion of COVID-19 infection among healthcare workers following both the first and second doses (Fig 5C).

### Characteristics of vaccine hesitant participants

**Fig 6:**
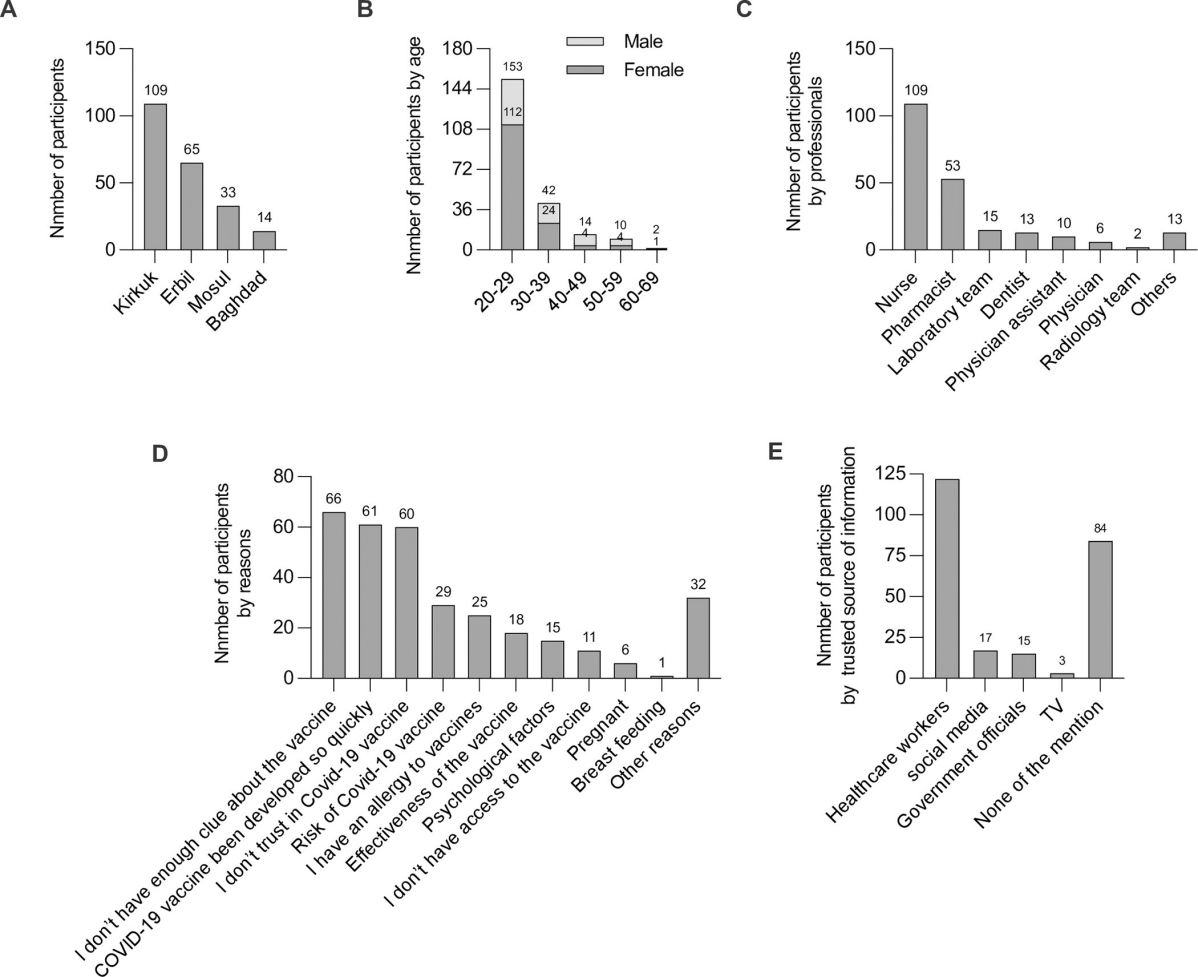
Characteristics of vaccine-hesitant participants. (A) Number of vaccine-hesitant participants by counties, (B) number of vaccine-hesitant participants by age groups, (C) number of vaccine-hesitant participants by professionals, (D) number of vaccine-hesitant participants based on reasons of rejecting the vaccines, (E) number of vaccine-hesitant participants based on source-trusted information about the vaccines.

A total of 221 healthcare workers were hesitant to receive the COVID-19 vaccines. In our study, the prevalence of COVID-19 vaccination hesitancy was 10.03%. Females had higher vaccine hesitancy (63.8%) (141) than males (36.2%) (80). Vaccine-hesitated participants were from four counties: 109 in Kirkuk (49.3%), 65 in Erbil (29.4%), 33 in Mosul (14.9%), and 14 in Baghdad (6.4%) (Fig 6A). The participants were divided into five main groups based on their age. Most participants were younger than 30 years of age, 20–29 (69.2%), 30–39 years (19.1%), 40–49 years (6.3%), 50–59 years (4.5%), and 60–69 years (0.9%). COVID-19 vaccination hesitancy decreased with increasing age (Fig 6B). The sample of vaccine-hesitant healthcare workers included several types of professionals; 109 nurses (49.3%), 53 pharmacists (23.9%), 15 laboratory team members (6.7%), 13 dentists (5.9%), 10 physician assistants (4.6%), 6 physicians (2.8%), 2 radiology team members (0.9%), and 13 staff members at healthcare facilities without direct patient contact (5.9%) (Fig 6C).

The next step was to determine the reasons for COVID-19 vaccination hesitancy. The three top reasons for rejecting the vaccine were insufficient knowledge about the vaccines (29.9%), COVID-19 vaccine was developed so quickly (27.6%), and there is no trust in the vaccine (27.1%) (Fig 6D). Other reasons for unwillingness to take the vaccine were risk of COVID-19 vaccine (13.1%), having an allergy to vaccines (11.3%), having concerns about the vaccine’s efficacy (8.1%), psychological factors (6.8%), having concerns about access to the vaccine (4.9%), pregnancy (2.7%), and breast feeding (0.4%). Of note, some participants chose more than one reason (Fig 6D). The majority of participants (48.4%) believed that healthcare professionals provided reliable information about the COVID-19 vaccine. The next most trusted sources were social media (7.6%), government officials (6.7%), and television (1.3%). However, 38% of the participants did not believe in any source of information about COVID-19 vaccines.

## Discussion

As vaccination plays a vital role in developing herd immunity, COVID-19 vaccines have reduced the incidence of COVID-19 infections around the world [25, 26]. Healthcare workers were the first people in Iraq to receive COVID-19 vaccines; professionals, including physicians, dentists, pharmacists, physician assistants, and nurses are thought to be directly affected by the pandemic’s critical health aspects due to their working environment and exposure to COVID-19 patients. In our study, over half of the participants (54%) reported that they dealt with COVID-19 patients frequently, 18.7% dealt occasionally, and 27.3% had no direct contact. However, no major study has assessed the adverse effects of COVID-19 vaccines in Iraq. Therefore, this study aimed to investigate the adverse effects associated with COVID-19 vaccines among healthcare workers in Iraq and to identify possible variations in symptoms initiated by different vaccines. Additionally, we aimed to assess the attitudes of vaccine-hesitant participants toward vaccination.

A cross-sectional study was conducted among healthcare professionals to ensure the accuracy and integrity of data collected to the best ability. It was crucial to assemble these data from trusted and expert sources that could provide accurate and transparent information. The Iraqi government authorized three different kinds of COVID-19 vaccines: the Pfizer-BioNTech mRNA vaccine (BNT162b2) (coding for SARS-CoV-2 spike protein), the Oxford-AstraZeneca vaccine (ChAdOx1 nCoV-19) (a replication-deficient chimpanzee adenovirus particle), and the Sinopharm vaccine (BBIBP-CorV) (an inactivated strain of SARS-CoV-2).

At the time of completing the questionnaire, most of the participants had received two doses of COVID-19 vaccines. Several adverse effects were reported by the participants who received either one dose or two doses of COVID-19 vaccines, mainly including pain at the site of injection, muscle pain, headache, high temperature, joint pain, fatigue, tenderness, feeling being sick, lethargy, redness/ pruritus at the site of injection, feeling achy, cough, nausea, loss of taste or smell, and diarrhea. In the current study, the adverse effects reported were not severe, and none of the participants required admission to the hospital. This is a positive finding for the public, who are worried about the safety of COVID-19 vaccines.

The Pfizer vaccine had a significant impact on the immune response after the first dose, resulting in a higher incidence of adverse effects in compared to the second dose [27, 28]. However, fatigue and feeling being sick were significantly more prevalent after receiving the second dose of the Pfizer vaccine. This happens due to an enhanced immune system response with increased immunoglobulin binding to antigens and the generation of a virus-neutralizing response [29]. Andrzejczak et al., 2021 also showed that some adverse effects of the Pfizer vaccine were more prevalent following the second dose [3].

The Pfizer vaccine had no adverse effects on 13.7% of the participants who received their second dose and 10.4% of those who received their first dose. The prevalence of non-reported adverse effects from the second and first AstraZeneca doses was 29% and 10.8%, respectively. Whereas, 36.9% and 27.7% of participants who received the second and first doses, respectively, of the Sinopharm vaccine did not report any adverse effects. Based on these results, the Pfizer vaccine may result in a stronger immune response than the AstraZeneca vaccine or the Sinopharm vaccine. Vaccines are known to potentially cause adverse reactions depending on the type of vaccine [30].

Female participants reported significantly more adverse effects than male participants. Common adverse effects associated with Pfizer’s first and second doses were significantly more prevalent in females than in males, such as pain at the site of injection, muscle pain, high temperature, joint pain, fatigue, tenderness and feeling being sick. A study conducted in Spain also found that Pfizer vaccines were associated with more adverse effects among females than males [31].

However, there were no significant difference between males and females in the prevalence of the adverse effects reported after the first dose of AstraZeneca. This was the same for the second AstraZeneca dose, except for the increased prevalence of higher temperatures in women compared to men. In contrast to our study, a study conducted in Bangladesh reported that females had more common adverse effects than males after receiving the first AstraZeneca dose [32].

Pain at the site of injection after Sinopharm’s first dose was significantly higher in females than in males. Nonetheless, Sinopharm’s second dose was associated with a significantly higher prevalence of some adverse effects in females than in males, including pain at the site of injection, headache, joint pain, fatigue, and loss of taste or smell. This was emphasized in a study conducted by Saeed et al. (2021), which exhibited more common adverse effects in females compared to males [23]. Another study on the Sinopharm vaccine found that females suffered from more adverse effects than males, (55% and 45%, respectively) [33].

Pain and redness at the site of injection after the first dose were more prevalent in the Pfizer vaccine than in the AstraZeneca or Sinopharm vaccines. In line with our study, local injection site symptoms were the most common adverse effects in the Pfizer COVID-19 clinical trial [34]. Another study on healthcare workers in the Czech Republic also found that injection site pain was the most common adverse effect [7].

Participants who received AstraZeneca’s first dose had significantly more adverse effects, including muscle pain, headache, high temperature, joint pain, fatigue, feeling being sick, and feeling achy, than those who received the Pfizer or Sinopharm vaccines. It has been reported that individuals who have received the AstraZeneca vaccine are more likely to have systemic adverse effects, such as fatigue and fever, than those who have received the Pfizer vaccine [35]. However, following the second dose, the participants who received the Pfizer vaccine had significantly more adverse effects than those who received the AstraZeneca or Sinopharm vaccines. These results were consistent with a comparison study on the Pfizer and AstraZeneca vaccines conducted in Saudi Arabia [8]. Participants who received Pfizer’s second dose reported a significantly higher frequency of common adverse effects than those who received AstraZeneca or Sinopharm vaccines. This may be due to variations in vaccine types and the boosting of the immune system. In our study, the incidence of COVID-19 infections among healthcare workers was significantly reduced after receiving one dose or two doses of either Pfizer, AstraZeneca or Sinopharm vaccines. Consistent with a study conducted by Hall et al., 2021, we found that vaccination reduces the occurrence of COVID-19 infections by inducing an immune response against it [36].

Despite the availability of the COVID-19 vaccines for healthcare workers, there is a variation in the hesitancy level for receiving the vaccine. About 29% of New York residents thought they would refuse a vaccine, compared with 20% of Canadians and 6% of the United Kingdom [37, 38]. We found that 10.03% of the participants were hesitant to receive the vaccine, and that this was more prevalent in females than in males, as well as in younger age groups compared to older age groups. Female gender was associated with a significantly lower likelihood of intending to accept a COVID-19 vaccine in 35 studies [39]. Vaccine hesitancy is significantly associated with age, and its prevalence is higher among younger age groups [40]. The common reasons for rejection of COVID-19 vaccines were insufficient knowledge surrounding the vaccine and the rapid development of COVID-19 vaccines, which provoked the participant’s loss of trust in the vaccine. According to a study among people over the age of 18, the most common reason for refusing vaccines was that they undermined vaccine reliability and rapid development [41].

## Conclusions

Most of the participants reported common adverse effects associated with the COVID-19 vaccines but none of them required hospitalization. Pain at the injection site was the most common adverse effect for all three vaccines: Pfizer, AstraZeneca, and Sinopharm. After first dose, common adverse effects were documented more with the AstraZeneca vaccine, followed by Pfizer and then Sinopharm vaccines. After second dose, common adverse effects were documented more with the Pfizer vaccine, followed by AstraZeneca and then Sinopharm vaccines. Females were more likely to show symptoms than males after receiving the COVID-19 vaccines. However, COVID-19 infections were significantly reduced after vaccination, regardless of the vaccine type. Participants who received two doses of Pfizer vaccine were significantly less likely to have COVID-19 infection than those who received two doses of AstraZeneca or Sinopharm vaccines. Further follow-up studies are required to evaluate the effectiveness of the vaccines, their prevention of SARS-CoV-2 infection, and their long-term adverse effects.
